# Culture-Free Whole Genome Sequencing of *Mycobacterium tuberculosis* Using Ligand-Mediated Bead Enrichment Method

**DOI:** 10.1093/ofid/ofae320

**Published:** 2024-06-15

**Authors:** Shruthi Vasanthaiah, Renu Verma, Ajay Kumar, Aravind K Bandari, John George, Mona Rastogi, Gowrang Kasaba Manjunath, Jyoti Sharma, Abhishek Kumar, Janavi Subramani, Kiran Chawla, Akhilesh Pandey

**Affiliations:** Manipal Academy of Higher Education, Manipal, Karnataka, India; Institute of Bioinformatics, International Technology Park, Bangalore, Karnataka, India; Manipal Academy of Higher Education, Manipal, Karnataka, India; Institute of Bioinformatics, International Technology Park, Bangalore, Karnataka, India; Manipal Academy of Higher Education, Manipal, Karnataka, India; Manipal Academy of Higher Education, Manipal, Karnataka, India; Institute of Bioinformatics, International Technology Park, Bangalore, Karnataka, India; Manipal Academy of Higher Education, Manipal, Karnataka, India; Institute of Bioinformatics, International Technology Park, Bangalore, Karnataka, India; Department of Laboratory Medicine and Pathology, Center for Individualized Medicine, Mayo Clinic, Rochester, Minnesota, USA; Manipal Academy of Higher Education, Manipal, Karnataka, India; Institute of Bioinformatics, International Technology Park, Bangalore, Karnataka, India; Manipal Academy of Higher Education, Manipal, Karnataka, India; Institute of Bioinformatics, International Technology Park, Bangalore, Karnataka, India; Manipal Academy of Higher Education, Manipal, Karnataka, India; Institute of Bioinformatics, International Technology Park, Bangalore, Karnataka, India; Manipal Academy of Higher Education, Manipal, Karnataka, India; Institute of Bioinformatics, International Technology Park, Bangalore, Karnataka, India; Manipal Academy of Higher Education, Manipal, Karnataka, India; Department of Microbiology, Kasturba Medical College Manipal, Manipal Academy of Higher Education, Manipal, Karnataka, India; Manipal Academy of Higher Education, Manipal, Karnataka, India; Institute of Bioinformatics, International Technology Park, Bangalore, Karnataka, India; Department of Laboratory Medicine and Pathology, Center for Individualized Medicine, Mayo Clinic, Rochester, Minnesota, USA; Center for Molecular Medicine, National Institute of Mental Health and Neurosciences, Bangalore, Karnataka, India

**Keywords:** culture-free, drug resistance, *Mycobacterium tuberculosis*, TB beads, whole genome sequencing

## Abstract

**Background:**

Direct whole genome sequencing (WGS) of *Mycobacterium tuberculosis* (*Mtb*) can be used as a tool to study drug resistance, mixed infections, and within-host diversity. However, WGS is challenging to obtain from clinical samples due to low number of bacilli against a high background.

**Methods:**

We prospectively collected 34 samples (sputum, n = 17; bronchoalveolar lavage, n = 13; and pus, n = 4) from patients with active tuberculosis (TB). Prior to DNA extraction, we used a ligand-mediated magnetic bead method to enrich *Mtb* from clinical samples and performed WGS on Illumina platform.

**Results:**

*Mtb* was definitively identified based on WGS from 88.2% (30/34) of the samples, of which 35.3% (12/34) were smear negative. The overall median genome coverage was 15.2% (interquartile range [IQR], 7.7%–28.2%). There was a positive correlation between load of bacilli on smears and genome coverage (*P* < .001). We detected 58 genes listed in the World Health Organization mutation catalogue in each positive sample (median coverage, 85% [IQR, 61%–94%]), enabling the identification of mutations missed by routine diagnostics. Mutations causing resistance to rifampicin, isoniazid, streptomycin, and ethambutol were detected in 5 of 34 (14.7%) samples, including the *rpoB* S441A mutation that confers resistance to rifampicin, which is not covered by Xpert MTB/RIF.

**Conclusions:**

We demonstrate the feasibility of magnetic bead–based enrichment for culture-free WGS of *Mtb* from clinical specimens, including smear-negative samples. This approach can also be integrated with low-cost sequencing workflows such as targeted sequencing for rapid detection of *Mtb* and drug resistance.

## Graphical Abstract

**Graphical Abstract:**
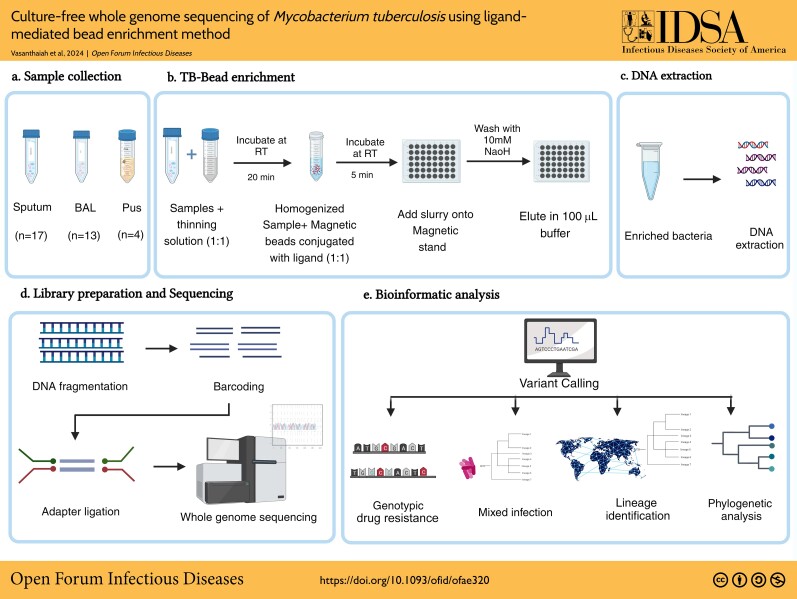
This graphical abstract is also available at Tidbit: https://tidbitapp.io/tidbits/culture-free-whole-genome-sequencing-of-mycobacterium-tuberculosis-using-ligand-mediated-bead-enrichment-method

Tuberculosis (TB) remains a major public health problem. As per the recent World Health Organization (WHO) report, there was a 3.9% increase in TB incidence rate between 2020 and 2022 [1]. India contributes to the world's highest TB incidence, with approximately 3 million cases annually [2]. Over the last decade, due to the accessibility of low-cost benchtop sequencers, streamlined workflows, and a faster turnaround time, whole genome sequencing (WGS) has rapidly evolved from a mere research tool to a clinical platform with the ability to identify the pathogen and its resistance to drugs [3], investigate transmission [4], and conduct public health surveillance [5–7]. However, WGS has not yet entered the mainstream of clinical diagnosis in TB, mainly due to the requirement of culturing *Mtb* prior to sequencing [7–9]. Unlike most laboratory procedures, culture-based *Mtb* analysis remains challenging due to slow growth of mycobacteria, laborious procedures, and expensive biocontainment facility requirement, precluding its use in resource-limited settings [10]. Additionally, in culture-based assays, typically a few single-colony subcultures are taken for the subsequent molecular analysis and sequencing. This may create a bias due to underrecognition of heterogeneity and mixed infections [8, 11–13]. Rapid molecular assays such as Xpert MTB/RIF (Cepheid, Sunnyvale, California) and GenoType MTBDRplus and MTBDRsl (Hain Life science GmbH, Germany) detect *Mtb* directly from clinical samples with a shorter turnaround time [14]. However, these assays are limited by the number of mutations they can target that can lead to false negatives, and do not provide information to investigate the pathogen genome [15].

Utilizing *Mtb* DNA isolated directly from clinical samples can not only be used as a rapid diagnostic tool, but genomic analysis of *Mtb* from clinical samples may also provide valuable insights into lineage prevalence, within-host diversity and evolution of drug resistance. However, WGS of *Mtb* from direct samples is challenging due to low bacterial load and poor recovery of *Mtb* during sample processing [9, 16]. In a metagenomic analysis, Doughty and colleagues provided the first proof of principle on WGS of *Mtb* from sputum samples without enrichment. They sequenced 8 positive sputum samples to detect *Mtb* and perform metagenomic analysis. However, despite high sputum bacillary load, the sequencing coverage achieved was very low and the samples could not be used for basic genomic analysis such as detection of resistance, mixed infection, and identifying novel mutations [17]. Brown and colleagues subsequently used biotinylated RNA baits to capture the *Mtb* DNA extracted from sputum samples, and accurately detected drug-resistant phenotypes [18]. Using RNA baits, Goig et al further sequenced *Mtb* directly from sputum samples and delineated the transmission clusters of TB [19]. Verma et al further used RNA baits to sequence *Mtb* from pooled surface swabs collected from prison cells occupied by patients with active TB to study transmission [20]. While these studies demonstrated the importance of sequencing the *Mtb* genome without culture, due to high cost, long genome capture protocol (∼48 hours), and requirement of highly trained personnel, capture assays are less scalable in resource-constrained settings. Thus, further studies are required to explore cost-effective and alternate enrichment methods that can be employed for WGS of *Mtb* directly from clinical samples.

Ligand-coated magnetic bead technology, which specifically enriches *Mtb* from clinical samples, has been previously used for concentrating *Mtb* for fluorescent microscopy and culture. It was found that magnetic bead–based methods significantly increased the sensitivity of microscopy and positivity of *Mtb* on culture [21, 22]. However, this bead-based *Mtb* enrichment method for WGS of clinical samples has not been explored. Also, studies on WGS of *Mtb* from other sample types than sputum have been lacking, and the sensitivity of enrichment in these samples is yet to be explored. This is especially important in the case of extrapulmonary TB infections and samples from pediatric patients where bacillary load in the sputum samples is either undetectable or absent [23–25].

In this study, we used a ligand-mediated magnetic bead strategy to enrich *Mtb* from sputum, bronchoalveolar lavage (BAL), and pus samples from patients with active TB, and performed WGS. We further compared our findings with routinely performed diagnostic tests including smear microscopy, Xpert MTB/RIF, and culture. We assessed the utility of further exploring this enrichment method in culture-free rapid diagnosis of *Mtb*, detection of drug resistance, mixed infections, and phylogeny.

## METHODS

### Patient Enrollment and Ethical Statement

We enrolled 34 patients with confirmed active TB at the Kasturba medical college, Manipal, India, between June and September 2018. All participants were aged ≥18 years and provided written consent prior to study initiation. The patients included in the study were enrolled based on their fit with the inclusion criteria, and a convenience sampling method was followed. Samples were collected from patients who provided informed consent. The study was approved by the institutional review board of Kasturba Medical College, Manipal Academy of Higher Education (IEC:526/2018).

### Sample Collection

We collected approximately 1 mL clinical specimen from active TB patients in replicates. This included early morning sputum (n = 17), BAL (n = 13), and pus (n = 4). BAL and pus samples were collected aseptically. One tube was used for the routine microbiological testing and reporting, and the other tube was used for magnetic bead enrichment and DNA extraction for sequencing. All samples were screened for *Mtb* bacilli using Ziehl-Neelsen staining and classified as scanty, 1+, 2+, or 3+ based on the Revised National Tuberculosis Control Program guidelines [26]. The samples were also tested on Xpert MTB/RIF (Cepheid, Sunnyvale, California) assay and inoculated on liquid (mycobacterial growth indicator test [MGIT]) and solid culture (Lowenstein-Jensen [LJ]) media. The *Mtb* growth was monitored for 45 days on MGIT and 8 weeks on LJ slants. Five sputum samples that were negative for *Mtb* on microscopy, Xpert MTB/RIF, and culture were taken as negative controls and enriched with magnetic beads followed by DNA extraction.

### Sample Processing

**Figure 1.**
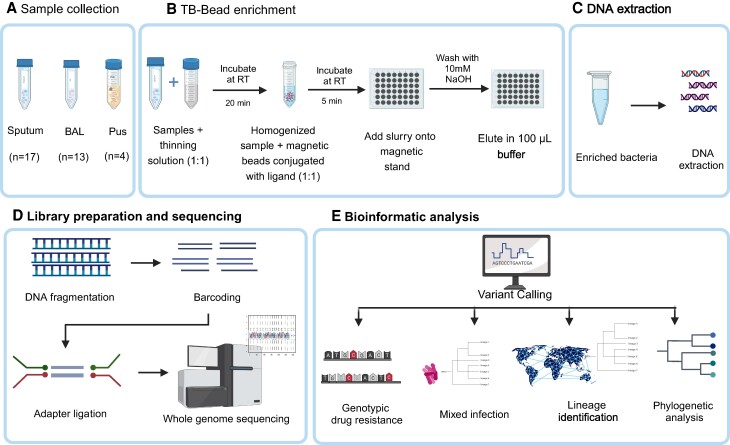
Workflow schema for bead-based *Mycobacterium tuberculosis* (*Mtb*) enrichment and whole genome sequencing: For bead concentration, sample is mixed with equal volume of thinning solution and homogenized after incubation. Slurry is mixed with magnetic beads conjugated with ligands to enrich *Mtb* cells and incubated for binding. The *Mtb* cells are eluted in 100 μL of elution buffer, which is subsequently used for DNA extraction. The extracted DNA is quality tested and used for library preparation using Nextera XT DNA Library Preparation Kit (Illumina). Bioinformatic analysis is performed to detect resistance, phylogeny, mixed infections, and new mutations. Abbreviations: BAL, bronchoalveolar lavage; NaOH, sodium hydroxide; RT, room temperature; TB, tuberculosis. Created with BioRender.com.

Samples were decontaminated before extraction by *N*-acetyl L-cystine–sodium citrate NaOH method. The beads were added in the concentrate obtained post–decontamination step. The samples collected were processed using TB-Bead kit (Microsens Diagnostics Ltd, London, United Kingdom) following the manufacturer's instructions in a Biosafety Level 3 (BSL-3) facility. In brief, the samples were thinned by adding equal volume of thinning solution and allowed to stand at room temperature for 20 minutes after mixing. The homogenized samples were mixed with equal volume of magnetic beads conjugated with the ligand. After mixing gently, the samples and the bead slurry were allowed to stand at room temperature for 5 minutes to capture the mycobacterial cells. The slurry was then placed on a magnetic stand to separate, and supernatant was discarded. The beads were washed with 10 mM NaOH in quick succession prior to the elution of the bound mycobacteria from the beads using a 100-μL elution buffer. Eluate containing enriched mycobacteria was used for DNA extractions. Workflow schema for bead enrichment and WGS is shown in Figure 1.

#### DNA Extraction and Library Preparation

The eluate enriched for mycobacteria was homogenized using the MP Biomedical bead beater (MP Biomedicals) and DNA was extracted using QIAamp DNA Mini Kit, according to the manufacturer's instructions. The DNA eluted from the column in 10 mM Tris buffer was stored at −80°C until further use. Isolated genomic DNA was quantified using qubit fluorometric method. Indexed libraries were prepared for each of the samples using a low-input genomic DNA library preparation kit (Nextera XT DNA Library Preparation Kit, Illumina) as per the manufacturer's instructions. In brief, DNA isolated from the samples was fragmented and the DNA fragments obtained were subjected to end-repair before the ligation of indexed adapter sequences. The indexed libraries were further amplified using polymerase chain reaction (PCR) to obtain the required amount of indexed library from each sample considering the low DNA yield. Qualities of the libraries were assessed using Agilent Bio Analyzer before proceeding with the clustering and sequencing process. The clustered libraries were sequenced on Illumina HiSeq XTen platform to obtain 150 bp paired-end reads.

#### Whole Genome Sequencing and Data Acquisition

About 0.5 GB data were generated per sample using the high output mode, to provide a depth of about 100 times for the microbial genome. Quality of the acquired data in FastQ file formats was assessed using FastQC (v0.11.5) and processing of the raw reads was carried out using FastQC fqtrim (v0.9.4) [27] tools. We trimmed low-quality bases (Phred-scaled base quality <20 were discarded). Quality processed reads were mapped against “Mycobacteria” (Taxonomy ID: 1763) sequences using Bowtie2 (v2.2.5) [28]. To rule out the DNA contamination from possible host organisms, we aligned raw reads against the reference genome of human (*Homo sapiens*), build 39, downloaded from the National Center for Biotechnology Information (NCBI). To identify the pathogenic bacteria from the WGS, we aligned the processed reads against representative bacterial/archaeal genomes database available at NCBI, using Bowtie2 with aforementioned parameters. This database comprised of genome sequences from 4378 bacterial species

After performing quality control, the raw sequencing data were aligned against the complete genome of *Mtb* H37Rv (RefSeq Accession: NC_000962.3) using Burrows-Wheeler Aligner using default parameters [29]. PCR duplicates were removed and the reads mapping to the reference genome were fetched and counted using Samtools (https://github.com/samtools/) [30]. A total of 336 complete genomes of different *Mycobacterium* species were downloaded from NCBI, which includes the available complete genomes of different strains of *Mtb*. All mapped reads were aligned to these complete *Mycobacterium* genomes using blastn program, and the reads that were matching with 100% identity were fetched. Furthermore, reads that are unique to H37Rv were fetched using a Python program.

### Variant Calling, Drug Resistance, and Phylogenetic Analysis

Variant calling was performed using of bcftools (version 1.18.) [30]. We used TB-Profiler [31] tool to carry out the identification of mutations associated with anti-TB drug resistance. We compared the list of mutations derived from TB-Profiler with the comprehensive list provided by the WHO for the association of drug resistance [32]. We also performed phylogenetic analysis of enriched samples using TB-Profiler to classify samples into lineages and sublineages and detect mixed infections. Additionally, for variant calling quality control, we considered only those mutations that had a minimum depth of 20× as per Illumina WGS minimum variant calling cutoff (10×) [33]. Tablet graphical viewer (v1.21.02.08) was used to visualize the genome location, lineage, and depth at that position supporting mixed infections. We used the MAFFT tool (v7) to generate multiple sequence alignment files and constructed a phylogenetic tree using RAxML-NG (v0.9.0).

### Statistical Analysis

We performed Kruskal-Wallis test to determine if there is a significant difference in the genome coverage based on smear grading. Samples were divided into negative, scanty, 1+, 2+, or 3+ based on smear grading, and *P* values were calculated. We also performed Kruskal-Wallis test to determine if the percentage of reads that mapped to the H37Rv genome varied based on the sample type. All analyses were performed in R software (v4.3.1).

## RESULTS

### Patient Characteristics

A total 34 individuals with confirmed active TB were recruited for the study. Among these, 73.5% (25/34) of the participants were male (median age, 49 years [interquartile range {IQR}, 33.5 years]). Median age of the female participants was 39 years (IQR, 21.5 years). The samples consisted of sputum (n = 17), BAL (n = 13), and pus (n = 4). Of 34 samples, 64.7% were positive on smear microscopy. Among those, the samples were acid-fast bacilli (AFB) negative (n = 10), scanty bacilli (n = 4), AFB 1+ (n = 7), AFB 2+ (n = 6), and AFB 3+ (n = 3). A total of 76.5% of the samples were positive for *Mtb* growth on MGIT and LJ culture, and 91.2% samples were positive on Xpert MTB/RIF (Table 1). We did not observe any *Mtb* growth on culture or amplification in PCR for negative controls. Supplementary Table 1 describes sequencing quality and statistics for all samples including smear microscopy status, GeneXpert results, lineage distribution across samples and drug resistance profiles.

**Table 1.** Clinical and Microbiological Characteristics of 30 Samples That Passed Sequencing Quality Filter

Clinical Characteristic	Total (n = 30)^a^	BAL (n = 11)	Pus (n = 3)	Sputum (n = 16)
No of patients	30 (100)	11 (36.7)	3 (10)	16 (53.3)
Male sex	22 (73.3)	6 (54.5)	1 (33.3)	15 (93.8)
Age, y, median (IQR)	44.5 (29.3–57.8)	44 (27.5–47.5)	50 (44.5–61.5)	50 (32.8–63)
Smear grade at baseline				
3+	3 (10)	2 (18.2)	0 (0)	1 (6.3)
2+	6 (20)	1 (9)	0 (0)	5 (31.3)
1+	7 (23.3)	0 (0)	0 (0)	7 (43.8)
Scanty	4 (13.3)	2 (18.2)	0 (0)	2 (12.5)
Negative	10 (33.3)	6 (54.5)	3 (75)	1 (6.3)
Xpert MTB/RIF				
Positive	27 (90)	11 (100)	3 (100)	13 (81.3)
Negative	3 (10)	0 (0)	0 (0)	3 (18.7)
Culture (MGIT)				
Positive	23 (76.7)	6 (54.5)	1 (33.3)	16 (100)
Negative	7 (23.3)	5 (45.4)	2 (66.7)	0 (0)

Data are presented as No. (%) unless otherwise indicated.

Abbreviations: BAL, bronchoalveolar lavage; IQR, interquartile range; MGIT, mycobacterial growth indicator tube; PCR, polymerase chain reaction.

^a^Microbiologically positive sample is defined as a patient with presumptive tuberculosis (TB) with biological specimen positive for acid-fast bacilli, or positive for *Mycobacterium tuberculosis* on culture, or positive for TB through quality-assured rapid diagnostic molecular test.

### Whole Genome Sequencing of Bead-Enriched *Mtb*

**Figure 2.**
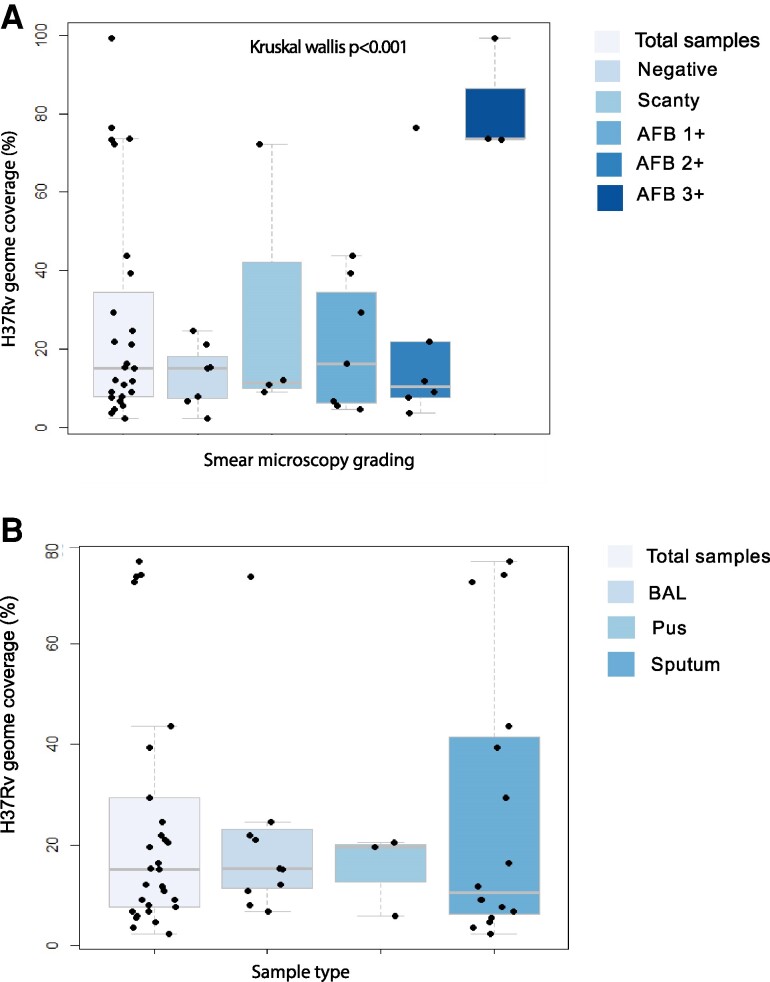
*A*, H37Rv genome coverage vs smear microscopy grading: There was a significant difference in percentage of H37Rv genome coverage among samples with high vs low smear positivity (*P* < .001). *B*, H37Rv genome coverage (%) vs sample type: The median H37Rv genome coverage in bronchoalveolar lavage (BAL), pus, and sputum was 10.5% 15.4%, and 19.6%, respectively. Of the total number of reads, the percentage of reads mapped to H37Rv genome were significantly low in sputum samples compared with BAL and pus. Abbreviations: AFB, acid-fast bacilli; BAL, bronchoalveolar lavage.

*Mtb* reads were detected in the majority of the samples (30/34 [88.2%]) with 35.3% (12/34) samples being smear negative. Four samples that could not be sequenced were positive for *Mtb* on Xpert MTB/RIF. However, they had low bacillary load indicated by smear microscopy. Two samples were smear negative: 1 was scanty while the other was AFB 1+. The total number of *Mtb*-specific reads detected in 30 samples that passed the quality cutoff was 3 831 740 (median reads per sample, 80 673). The samples had a median percentage genome coverage 15.2% (IQR, 7.7%–28.2%). There was a significant difference in percentage of H37Rv genome coverage among samples with high versus low smear positivity (Kruskal-Wallis *P* < .001; Figure 2*A*). Sequencing statistics based on various sample types is provided in Table 2.

**Table 2.** Sequencing Statistics for the Quality Filtered Samples Enriched Using Ligand-Based Magnetic Beads

Category	Sample Type			
	Total Samples (n = 30)	BAL (n = 11)	Sputum (n = 16)	Pus (n = 3)
Mean baseQ, median (IQR)	39.4 (38.7–39.8)	39.5 (39.3–39.9)	38.9 (38.6–39.4)	39.9 (39.85–40)
No. of reads mapped to H37Rv, median (IQR)	80 673 (46 293.5–119 590.5)	114 978 (78 407–171 945)	57 996 (35 128–84 677)	205 084 (148 304–208 652)
H37Rv genome coverage, %, median (min, max)	15.2 (7.7, 28.2)	15.4 (11.4, 23.2)	10.5 (6.5, 40.5)	19.6 (12.7, 20)
H37Rv genome coverage depth, ×, median (min, max)	0.63 (0.4, 1.1)	0.63 (0.44, 1.28)	0.6 (0.3, 0.9)	1.1 (0.6, 0.8)

Abbreviations: BAL, bronchoalveolar lavage; IQR, interquartile range.

Despite low bacillary count as per AFB staining, BAL and pus samples had higher *Mtb* genome coverage than sputum samples. The number of smear-negative samples in sputum, BAL, and pus was 1/17, 8/13, and 3/4, respectively. The median H37Rv genome coverage in sputum, BAL, and pus was 10.5% 15.4%, and 19.6%, respectively (Figure 2*B*). Of the total number of reads, the percentage of reads mapped to H37Rv genome were significantly low in sputum samples when compared with BAL and pus (*P* = .0147) (Supplementary Figures 1*A* and 1*B*). It has been previously observed that pus samples due to low *Mtb* load are difficult to culture for subsequent analysis [34]. This makes it challenging to analyze these samples on a comprehensive drug susceptibility testing (DST) panel or perform any genomic analysis. We observed that due to less complexity of BAL and pus samples, the overall bead enrichment yield was higher for these samples allowing WGS.

### Drug Resistance Detection

We compared variant calling data obtained from WGS of enriched samples with 58 genes from *Mtb* genome listed by the WHO as genes associated with drug resistance [32]. We detected reads mapping to these genes in all the samples with median percentage coverage 85% (IQR, 61%–94%) (Supplementary Table 2*A*).

**Figure 3.**
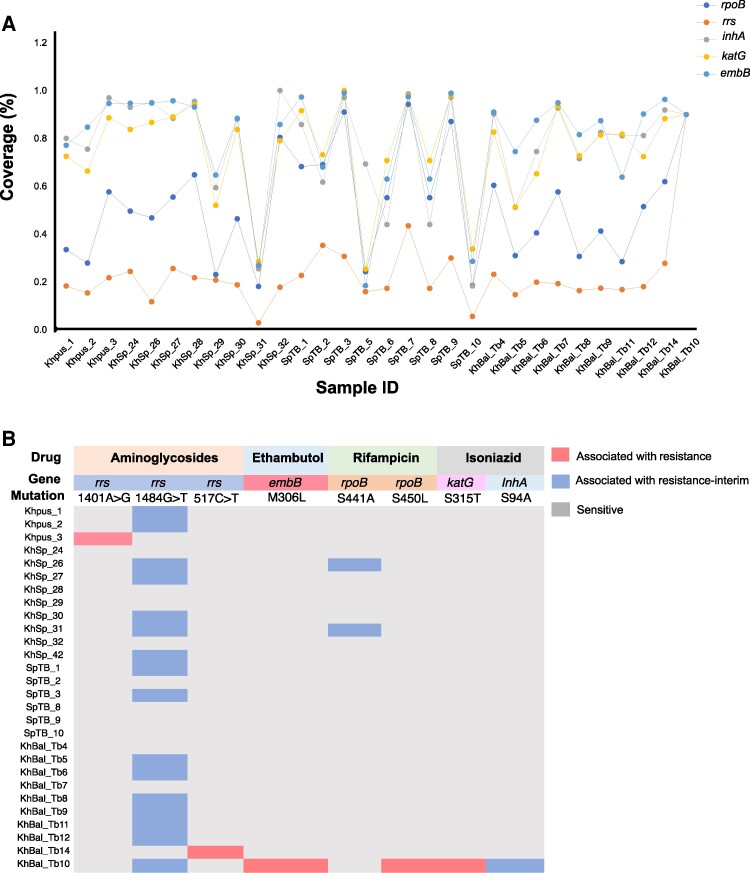
*A*, Percentage coverage of genes carrying mutations conferring drug resistance detected in 21 samples. The coverage trend per gene was similar in most of the isolates, with the *rrs* gene having the lowest and *embB* having the highest coverage. Reads mapping to these genes were detected in 99.9% of the samples, with median percentage coverage 85% (interquartile range, 61%–94%) *B*, Mutation trend in drug resistance genes: The majority of samples had mutation in *rrs* gene causing resistance to aminoglycosides. Mutations were classified as associated with resistance and associated with resistance interim as per the World Health Organization mutation catalogue. Samples with multiple mutations causing resistance to >1 drug were also observed.

We identified nonsynonymous mutations and mutations in noncoding exon within the genes that are known to play a role in causing resistance to anti-TB drugs when mutated. Among all the resistance-associated genes analyzed, we detected 8 different mutations in 5 genes (*rpoB*, *katG*, *rrs*, *inhA*, *and embB*) that confer resistance to first- and second-line anti-TB drugs in 18 of 30 (60.0%) samples. The H37Rv percentage genome coverage for these genes is shown in Figure 3*A*. We identified 5 different mutations in the *rrs* gene (1401A > G, 1484G > T, 1402C > A, 799C > T, and 888G > A). Of these, only 2 mutations, 1401A > G and 1484G > T, have been identified as mutations associated with resistance, while the remaining 3 have been listed as mutations of uncertain significance as per the recent WHO catalogue. A list of mutations in the genes associated with drug resistance that were detected in this study is provided in Supplementary Table 2*B*. We observed the 1484G > T mutation in the *rrs* gene in 15 samples. This mutation is known to confer interim resistance to aminoglycosides [35] and is reported to be highly prevalent in India [36]. Additionally, we observed the 1401A > G mutation in the *rrs* gene in 1 sample. This mutation is strongly associated with resistance to aminoglycosides [37] (Figure 3*B*).

S450L mutation in the *rpoB* gene that confers high-level resistance to rifampicin was detected in KhBal_Tb10 sample previously detected as rifampicin resistant on Xpert MTB/RIF. In addition to confirming rifampicin resistance, we also detected mutation in *katG* (Ser315Thr), *inhA* (Ser94Ala), and *rrs* (1484G > T) conferring resistance to isoniazid and aminoglycosides in KhBal_Tb10 sample. Mutations associated with ethambutol, streptomycin, and ethionamide were also detected in this same sample. KhBal_Tb10 was classified as pre–extensively drug resistant as per our WGS analysis. We detected a nonsynonymous mutation in *rpoB* gene (Ser441Ala) in 2 samples that were classified as rifampicin sensitive by Xpert MTB/RIF. However, the Ser441Ala *rpoB* mutation has been classified as an important mutation causing rifampicin interim resistance as per the WHO catalogue. In the AFB 2+ sample, the mutation frequency for Ser441Ala was 100%, with a coverage depth of 42×. The AFB3+ sample exhibited a mutation frequency of 87% at the Ser441Ala position, with a coverage depth of 235×.

It should be noted that while GeneXpert Ultra is very sensitive toward detecting rifampicin resistance and covers the “441” *rpoB* RRDR region position, the detection of mutations in GeneXpert Ultra is based on high-resolution melt curve analysis using specific molecular beacon probes, which detect specific melting temperatures for each mutation. The probes included in the GeneXpert Ultra are designed to detect only Ser441Gln and Ser441Leu mutations, and the Tm range required to detect the *rpoB*_S441A mutation is not part of the detection system [38].

### Phylogenetic Analysis and Mixed Infection Detection

Of the 30 samples, phylogenetic analysis successfully assigned lineages and sublineages to 9 samples (30%). This included BAL (n = 3), pus (n = 3), and sputum (n = 3) samples. The predominant lineage among these samples was lineage 1 (Indo-Oceanic), which was detected in 7 of 9 samples (77.8%). These findings are in concordance with the previously reported prevalence of *Mtb* lineages in India. Indo-Oceanic lineage is one of the major widespread lineages prevalent in specific geographical regions and is known to have high mortality rate [39]. Other lineages that were identified included lineages 4, 6, 7, and 9. A list of samples and their corresponding lineages identified by TB-Profiler, and the number of reads supporting each lineage, is provided in Table 3. Supplementary Figure 2 contains snapshots of the genomes from Tablet genome viewer where mixed infections were detected by TB-Profiler. The figure provides the genome location, lineage, and depth at that position.

**Table 3.** List of *Mycobacterium tuberculosis* Lineages in Various Samples Predicted on TB-Profiler Using Whole Genome Sequencing Data

Sample ID	Sample Type	Lineage	Sublineage	No. of Reads Supporting the Lineage (Phred Score ≥20)
KHBal-10	BAL	Lineage 1	…	215
		Lineage 1	1.2.2	183
		Lineage 1	1.2.2.2	211
KHBAl-8	BAL	Lineage 4	4.6.2.1	23
		Lineage 1	1.2.1.2	43
KHBAL_5	BAL	Lineage 9	…	26
		Lineage 7	…	20
		Lineage 4	4.6.3	22
		Lineage 4	4.1.1.3	20
		Lineage 1	1.2.1.2.1	22
Khpus_3	Pus	Lineage 1	1.1.1	51
Khpus_2	Pus	Lineage 4	4.6.2	22
		Lineage 6	6.3.1	30
Khpus_1	Pus	Lineage 6	6.3.1	30
KhSp_31	Sputum	Lineage 1	…	28
		Lineage 4	4.9	389
KhSp_27	Sputum	Lineage 1	1.1.1	21
KhSp_26	Sputum	Lineage 1	1.1.1	60

Abbreviation: BAL, bronchoalveolar lavage.

We observed a high frequency of mixed infection in BAL and pus samples when compared with sputum. Overall, mixed infections were detected in 4 samples (BAL, 2; pus, 1; and sputum, 1). KHPus_2 was infected with lineages 4 and 6. KHBAL_5 was infected with lineages 9, 7, 4, and 1. Although we used stringent criteria and a globally validated TB-Profiler tool for lineage identification, detecting mixed infections with multiple strains is rare. Further studies are required on a larger cohort to confirm these findings. Higher background and low sample quality of sputum samples may explain the nondetection of strains with low copy number. These findings are in line with higher median percentage genome coverage of BAL (median, 15.4%) and pus (median, 19.6%) samples than sputum (10.5%) despite low bacilli count as per smear microscopy.

## DISCUSSION

Due to significant advancement in WGS technologies in recent years, there has been an increase in accessibility to cost-effective sequencing platforms. However, the majority of sequencing studies use *Mtb* DNA extracted from cultures, which takes several weeks to months and requires a BSL-3 facility. Moreover, WGS sequencing from cultures may create a bias due to DNA extracted from selected colonies and may not detect mixed infections [8, 40]. Direct WGS from clinical samples could be a solution to eliminate these shortcomings; however, without any enrichment, it provides poor coverage that cannot be used for further analysis. To address these gaps, we used ligand-based magnetic beads to enrich *Mtb* from clinical samples and performed WGS. To our knowledge, this is the first study demonstrating the use of magnetic beads to enrich *Mtb* from clinical samples for WGS and direct sequencing of smear negative and extrapulmonary TB samples .We demonstrated that concentrating *Mtb* prior to DNA extraction significantly increased the sequencing positivity in the smear-negative samples. We were able to detect *Mtb*-specific reads in the majority (88.2%) of the samples, with 35.3% smear negative. In addition to confirming the drug resistance detected in routine diagnostics, we also identified mutations that confer resistance to other anti-TB drugs. These mutations are generally not covered by PCR-based tests due to limitations in the number of targets that can be screened. Direct WGS sequencing also allowed detection of mixed infections and classification of *Mtb* into respective lineages and sublineages in the majority of the samples (70%).

While we did not sequence unenriched samples to compare *Mtb* recovery with and without enrichment due to the limited sample quantity, our findings are in line with previously published studies. These studies compared the efficacy of using magnetic beads for *Mtb* enrichment for microscopy and culture and reported an overall improvement in the sensitivity after magnetic bead enrichment. Wang and colleagues observed significant improvement in microscopy sensitivity in enriched samples when compared with direct smear microscopy [41]. An increase in case finding (38%) was observed when bead-enriched sputum samples were used compared to direct smear microscopy [42]. Additionally, in a previous study, Doughty and colleagues sequenced 8 AFB 3+ sputum samples without any enrichment and observed a maximum 0.7× coverage depth [17]. Using magnetic bead enrichment, we obtained up to 18.7× coverage for AFB 3+ sample, suggesting improved sequencing coverage with magnetic bead enrichment. In addition to sequencing *Mtb* enriched from sputum, we also sequenced *Mtb* from BAL and pus samples, for which no direct sequencing data have been available thus far. It is interesting to note that pus and BAL samples that were negative on smear microscopy had relatively higher coverage than the smear-positive sputum samples. This could be explained due to high viscosity of sputum samples that might lead to trapping *Mtb* in the sputum inhibiting binding to the beads. An additional sputum thinning step may further improve the yield. Also, presence of other bacteria that would potentially bind to the beads and that cause saturation may also affect the yield. Further studies on a larger sample size are needed to confirm these findings.

The WHO has emphasized prioritizing the affordable and accessible point-of-care TB diagnostics, including for DST. As the WHO now recommends sequencing-based assays for *Mtb* drug resistance determinants, the relevant equipment and expertise may be increasingly available in public health laboratories in tuberculosis-endemic settings [1]. Currently available molecular assays target markers for a limited number of drugs and are not usually designed to incorporate a growing list of resistance mutations for additional drugs [43]. These assays can sometimes lead to false-negative results or underrepresentation of mutations in a population. Although diagnostics such as the Fluorotype MTB test (Hain, Germany) and the Anyplex II MTB/MDR can detect resistance to fluoroquinolones and second-line injectable drugs, their sensitivity ranges from 69.1% to 99.2% [44–46]. In a study to evaluate *Mtb* clinical isolates from Congo, it was found that there was a misidentification of fluoroquinolone resistance by line probe assay due to a double substitution T80A-A90G in *gyrA* gene [47]. Furthermore, additional assays are currently needed for surveillance or outbreak detection, adding to the overall cost. Therefore, developing a method that allows large-scale screening of mutations directly from clinical samples is an attractive prospect. We compared the WGS data obtained from our study with a comprehensive list of mutations published by the WHO that are either associated with or potentially linked to resistance against anti-TB drugs. In addition to confirming mutations detected on Xpert MTB/RIF, we detected a nonsynonymous Ser441Ala mutation in *rpoB* gene in 2 samples not covered by routine diagnostic assays. This mutation has been classified as a mutation causing interim resistance to rifampicin. Individuals carrying such mutations can benefit from dose adjustments. One such example is patients with mutations in *katG* and *inhA* genes. Mutations in *katG* confer high-level isoniazid resistance, whereas mutations in *inhA* causes low-level resistance to isoniazid [48]. Studies have shown that frequency of these mutations varies geographically and only 10% of the population has both *katG* and *inhA* mutations [49]. Therefore, patients with only *inhA* mutation may benefit from high doses of isoniazid [50]. Identifying the resistance and the frequency patterns of such mutations may help in making decisions regarding drug regimen. We observed *rrs* gene mutations in 15 of 34 (44.1%) samples, with 1484G > T mutation being the most prevalent. This mutation has been previously reported to be highly prevalent in India [36]. High occurrence of *rrs* gene mutations has been reported by several studies. A study in southern China detected *rrs* gene mutations in 22.5% of the samples, with 1401A > G being the most prevalent mutation [51]. Cuevas-Córdoba and colleagues reported *rrs* gene mutations in 28% of their study cohort, with isolates having >1 mutation [52]. Another study from Pakistan found *rrs* mutations in 30% of their samples, with samples having up to 6 mutations in the *rrs* gene [53]. Although our findings are in line with previously reported studies, considering the overall low coverage of our samples, further studies with a larger sample size utilizing magnetic bead sequencing method would be required to validate our findings.

Another relevant application of direct WGS is the study of *Mtb* genetic diversity in sputum samples, which might better reflect the within-patient bacterial populations. So far, the extent to which culture reduces the actual *Mtb* genetic diversity remains unclear. Although we did not compare the extent to which genetic diversity is lost during culture, studies have reported loss of minority strains during culture. Using mycobacterial interspersed repetitive unit–variable number tandem repeat genotyping method, Wang et al reported that after the addition of a culture step, 66.7% of strains with polyclonal infections had converted into a single genotype [54]. Using ultradeep next-generation sequencing, Metcalfe and colleagues found that there was a significant dynamic loss of minor-variant resistant subpopulations after subculture [55]. It is also likely that additional diversity can be identified in settings where several lineages coexist and generate mixed infections. Various studies have observed polyclonal *Mtb* infections among different populations to range between 14% and 19% [56, 57]. In a recent study, Kargarpour Kamakoli and colleagues found that 53% of the study cohort had polyclonal infection among which 46% harbored heteroresistance [58]. Studies have reported cases of reinfection by a second *Mtb* strain and occasional infection with >1 strain, making it difficult to assign the accurate treatment regimen [11, 12]. Infection with multiple strains of *Mtb* can complicate the interpretation of DST results and the detection of epidemiological links [13]. Using direct sequencing on enriched samples, we detected mixed infection (26.6%) of the samples. It is interesting to note that infection with up to 4 strains of *Mtb* was detected in pus samples. Studies have shown that patients harboring multiple strains of *Mtb* have poor treatment outcomes [59]. We used TB-Profiler, a globally validated tool for lineage identification, and further selected only those samples where each lineage was supported by at least 20 reads. While we were bioinformatically cautious in reporting mixed infections, our findings have limitations as we did not culture the samples to definitively confirm the presence of these lineages and have low sample coverage. Further studies comparing the magnetic bead sequencing method with paired culture would be required to validate these findings.

The findings of our study are subject to several limitations. First, we only sequenced samples enriched with magnetic beads and did not have WGS data on samples without enrichment to quantify the loss of *Mtb* cells during DNA extraction. Furthermore, while we observed a significant difference in terms of percentage reads mapping to H37Rv when compared between sputum, BAL, and pus, we analyzed a limited number of pus samples (n = 4) and further studies on a larger sample size would be required to validate these findings. We did not sequence the samples from culture to compare the extent of genetic diversity that might be lost when DNA extracted from culture is used for WGS. We did not perform a culture DST comparison of the resistance data for the confirmation, although the resistant data are compared with the WHO mutation catalogue. Additionally, we only analyzed 3 AFB 3+ samples to demonstrate that a magnetic bead–based method can be used to enrich *Mtb* for WGS to achieve sufficient coverage for basic analysis. While coverage obtained for most of the samples in our study was better than previously reported studies and allowed for basic analysis, this technique still requires further optimization to perform complex WGS analyses that require higher coverage.

## CONCLUSIONS

Amid the growing evidence that WGS of *Mtb* can detect drug resistance, predict pathogen prevalence and evolution patterns, and identify mixed infections and coinfections, there is a need for platforms that enable the direct WGS of *Mtb* from clinical samples, eliminating bias. We used magnetic bead enrichment method to capture *Mtb* prior to DNA extraction to perform WGS. Our strategy can be used to perform WGS on smear-negative and difficult-to-culture extrapulmonary TB samples. The enriched samples can also be used to perform targeted genome sequencing to achieve higher coverage. This strategy would be especially useful in samples that are not detected by routine diagnostic assays due to low bacillary load. The low yield of bacterial DNA poses a significant challenge when sequencing direct samples. Enrichment methods like hybrid capture assays are expensive and impractical in resource-limited settings. Although magnetic bead enrichment yields low coverage for WGS, it can provide adequate *Mtb* DNA for targeted sequencing. WHO has recently endorsed targeted sequencing-based assays for drug resistance diagnosis. With an enrichment cost of approximately US$3 per sample, the bead method offers scalability and integration potential with current and future low-cost targeted sequencing-based diagnostics. Magnetic bead enrichment is easier to perform, is cost-effective, and can be scalable for use in settings where TB burden is greatest.

## Supplementary Data

Supplementary materials are available at *Open Forum Infectious Diseases* online. Consisting of data provided by the authors to benefit the reader, the posted materials are not copyedited and are the sole responsibility of the authors, so questions or comments should be addressed to the corresponding author.

## Supplementary Material

ofae320_Supplementary_Data

## Notes

***Acknowledgments.*** The authors acknowledge all of the technical staff and patients from Manipal Hospital for their cooperation during the study.

***Data availability*.** Data supporting the findings of this manuscript have been submitted to NCBI and can be accessed through the Sequence Read Archive submission portal with accession number PRJNA1052384 (https://submit.ncbi.nlm.nih.gov/subs/sra/SUB14001592/overview).

***Financial support*.** R. V. is supported by the Ramalingaswami Fellowship, Department of Biotechnology, Government of India (ID No. BT/HRD/35/02/2006).
