# Supplementary material for: Culture-Free Whole Genome Sequencing of Mycobacterium tuberculosis Using Ligand-Mediated Bead Enrichment Method
Source: Open Forum Infect Dis. 2024 Jun 15;11(7):ofae320. doi: 10.1093/ofid/ofae320 (PMC11218775; doi:10.1093/ofid/ofae320)

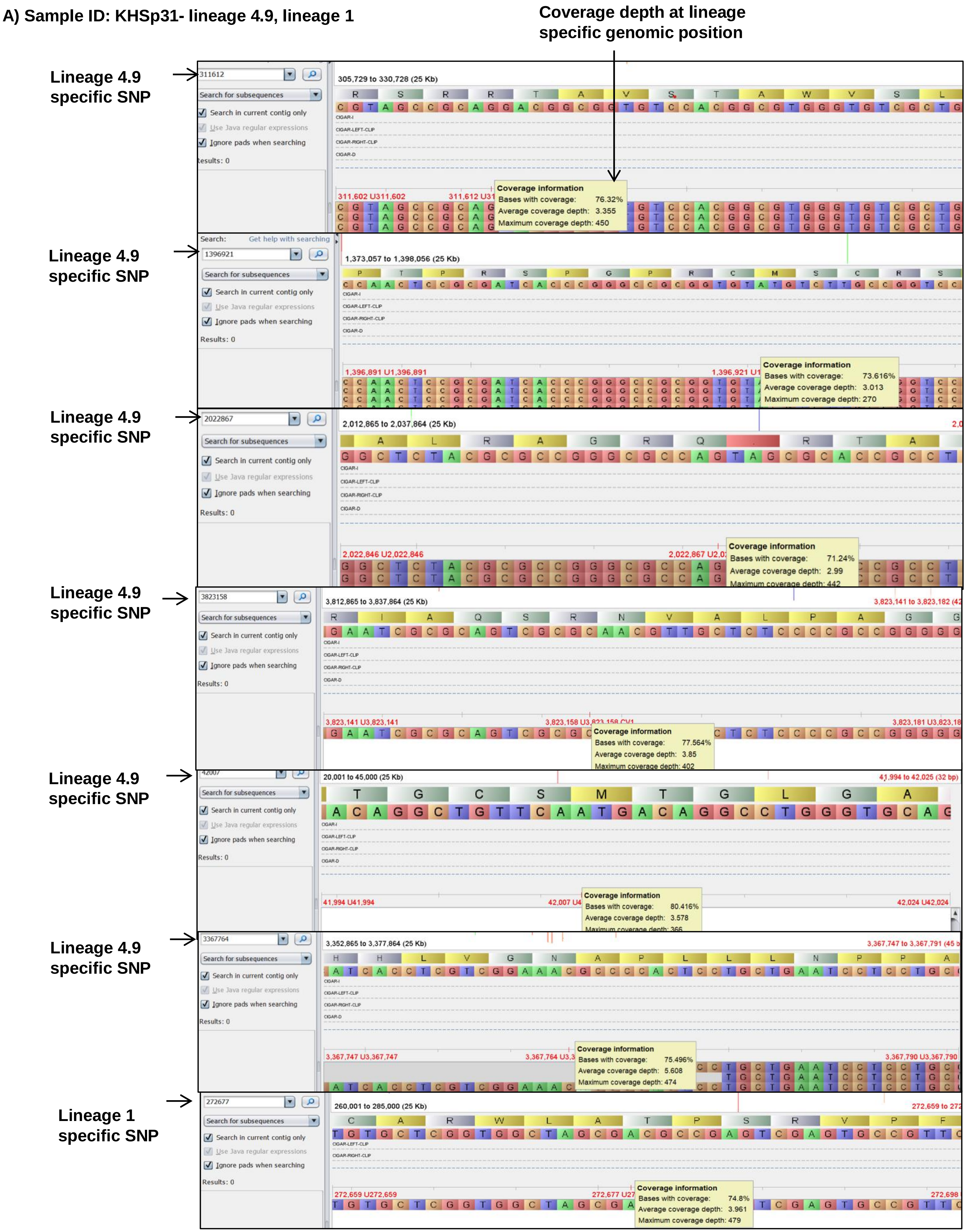

A) Sample ID: KHSp31- lineage 4.9, lineage 1
Coverage depth at lineage specific genomic position
Lineage 4.9 specific SNP
311612
305,729 to 330,728 (25 Kb)
Coverage information
Bases with coverage: 76.32%
Average coverage depth: 3.355
Maximum coverage depth: 450
Lineage 4.9 specific SNP
1396921
1,373,057 to 1,398,056 (25 Kb)
Coverage information
Bases with coverage: 73.616%
Average coverage depth: 3.013
Maximum coverage depth: 270
Lineage 4.9 specific SNP
2022867
2,012,865 to 2,037,864 (25 Kb)
Coverage information
Bases with coverage: 71.24%
Average coverage depth: 2.99
Maximum coverage depth: 442
Lineage 4.9 specific SNP
3823158
3,812,865 to 3,837,864 (25 Kb)
Coverage information
Bases with coverage: 77.564%
Average coverage depth: 3.85
Maximum coverage depth: 402
Lineage 4.9 specific SNP
20,001 to 45,000 (25 Kb)
Coverage information
Bases with coverage: 80.416%
Average coverage depth: 3.578
Lineage 4.9 specific SNP
3367764
3,352,865 to 3,377,864 (25 Kb)
Coverage information
Bases with coverage: 75.496%
Average coverage depth: 5.608
Maximum coverage depth: 474
Lineage 1 specific SNP
272677
260,001 to 285,000 (25 Kb)
Coverage information
Bases with coverage: 74.8%
Average coverage depth: 3.961
Maximum coverage depth: 479

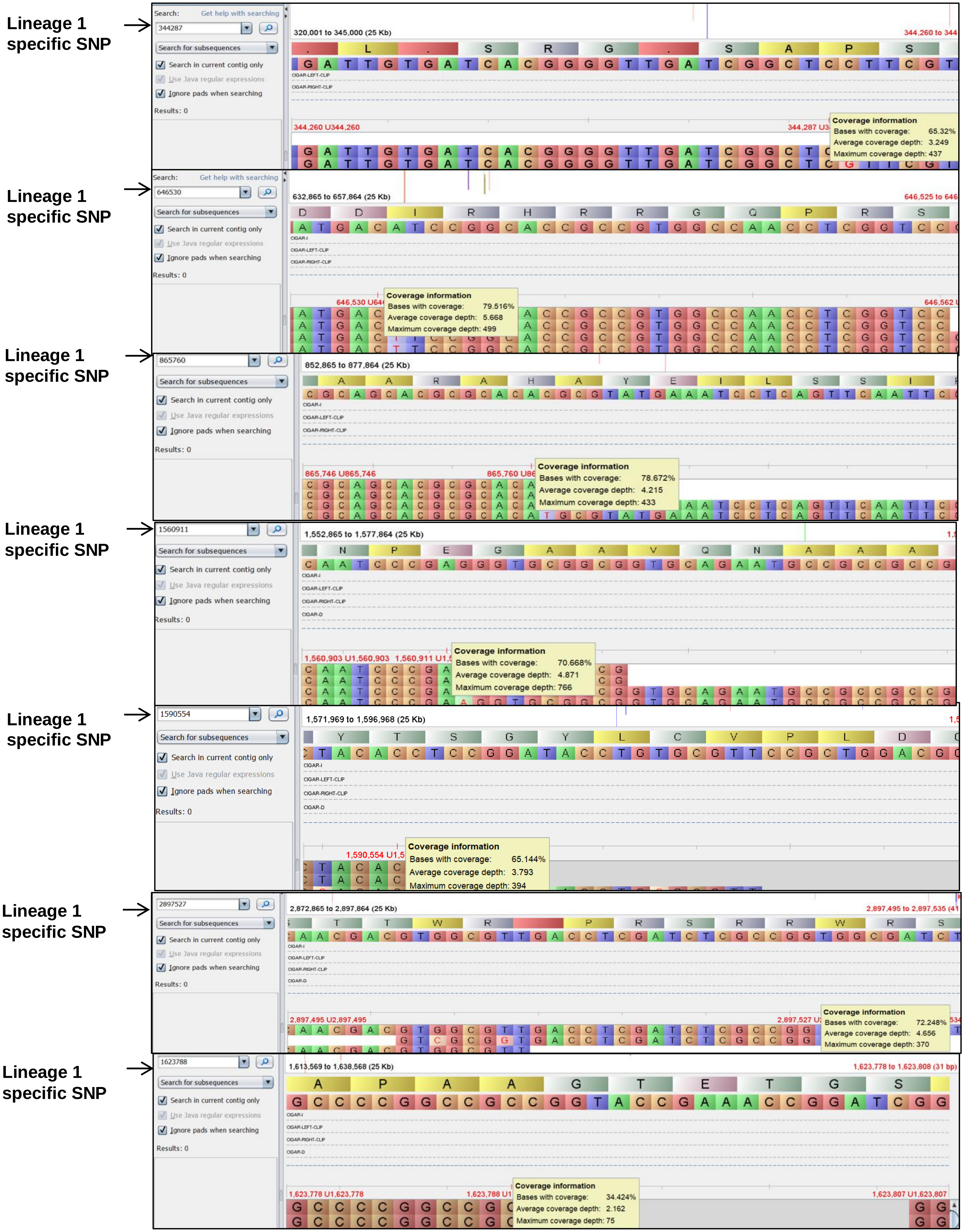

Lineage 1 specific SNP
344287
320,001 to 345,000 (25 Kb)
Coverage information
Bases with coverage: 65.32%
Average coverage depth: 3.249
Maximum coverage depth: 437
Lineage 1 specific SNP
646530
632,865 to 657,864 (25 Kb)
Coverage information
Bases with coverage: 79.516%
Average coverage depth: 5.668
Maximum coverage depth: 499
Lineage 1 specific SNP
865760
852,865 to 877,864 (25 Kb)
Coverage information
Bases with coverage: 78.672%
Average coverage depth: 4.215
Maximum coverage depth: 433
Lineage 1 specific SNP
1560911
1,552,865 to 1,577,864 (25 Kb)
Coverage information
Bases with coverage: 70.668%
Average coverage depth: 4.871
Maximum coverage depth: 766
Lineage 1 specific SNP
1590554
1,571,969 to 1,596,968 (25 Kb)
Coverage information
Bases with coverage: 65.144%
Average coverage depth: 3.793
Maximum coverage depth: 394
Lineage 1 specific SNP
2897527
2,872,865 to 2,897,864 (25 Kb)
Coverage information
Bases with coverage: 72.248%
Average coverage depth: 4.656
Maximum coverage depth: 370
Lineage 1 specific SNP
1623788
1,613,569 to 1,638,568 (25 Kb)
1,623,778 to 1,623,808 (31 bp)
Coverage information
Bases with coverage: 34.424%
Average coverage depth: 2.162
Maximum coverage depth: 75

B) Sample ID: KHBAL5- lineage 9, lineage 7, lineage 4.1.1.3, lineage 4.6.3, lineage 1.2.1.2.1

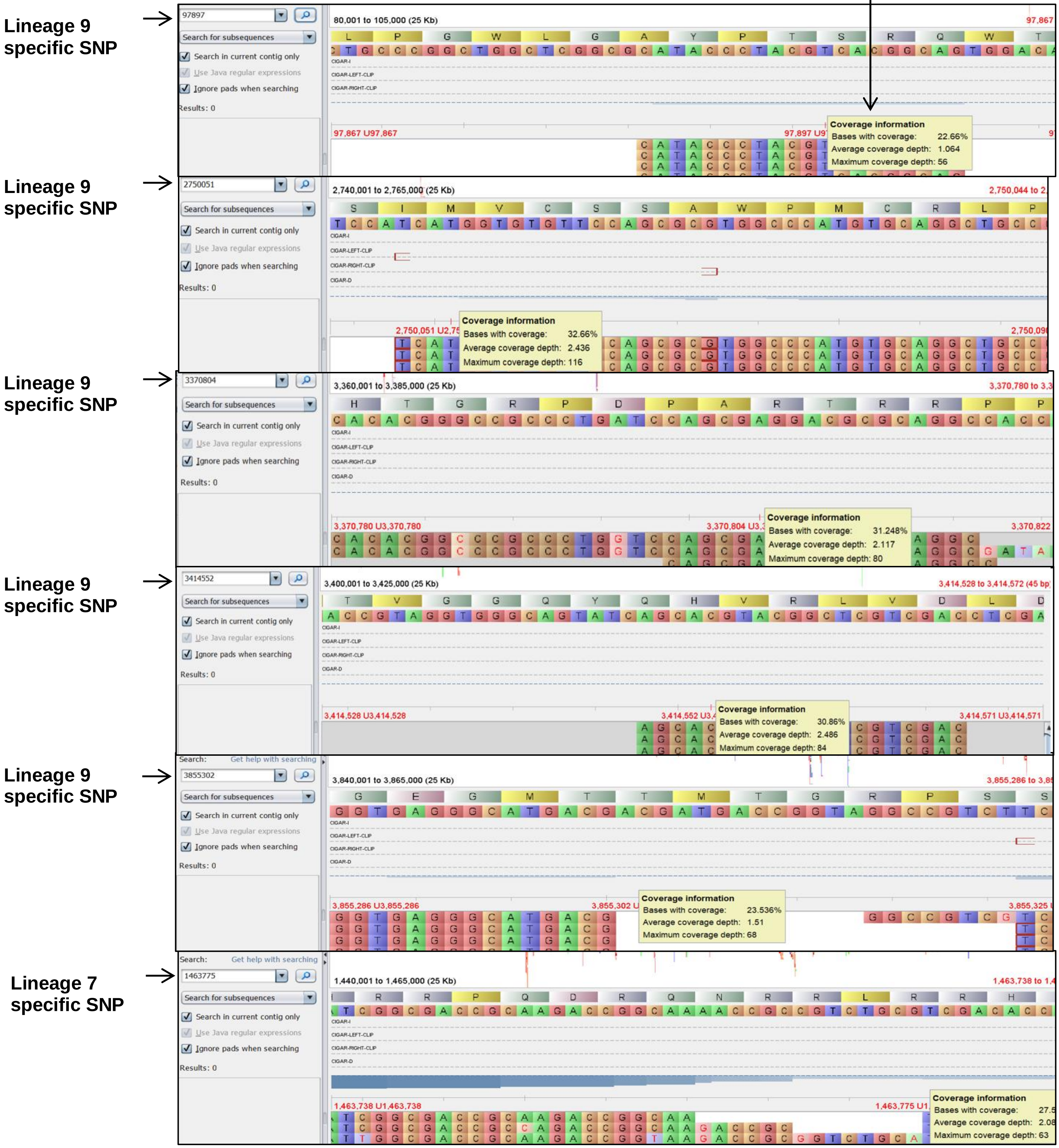

Lineage 4.6.3 specific SNP →

Lineage 4.1.1.3 specific SNP →

Lineage 4.1.1.3 specific SNP →

Lineage 1.2.1.2.1 specific SNP →

Lineage 1.2.1.2.1 specific SNP →

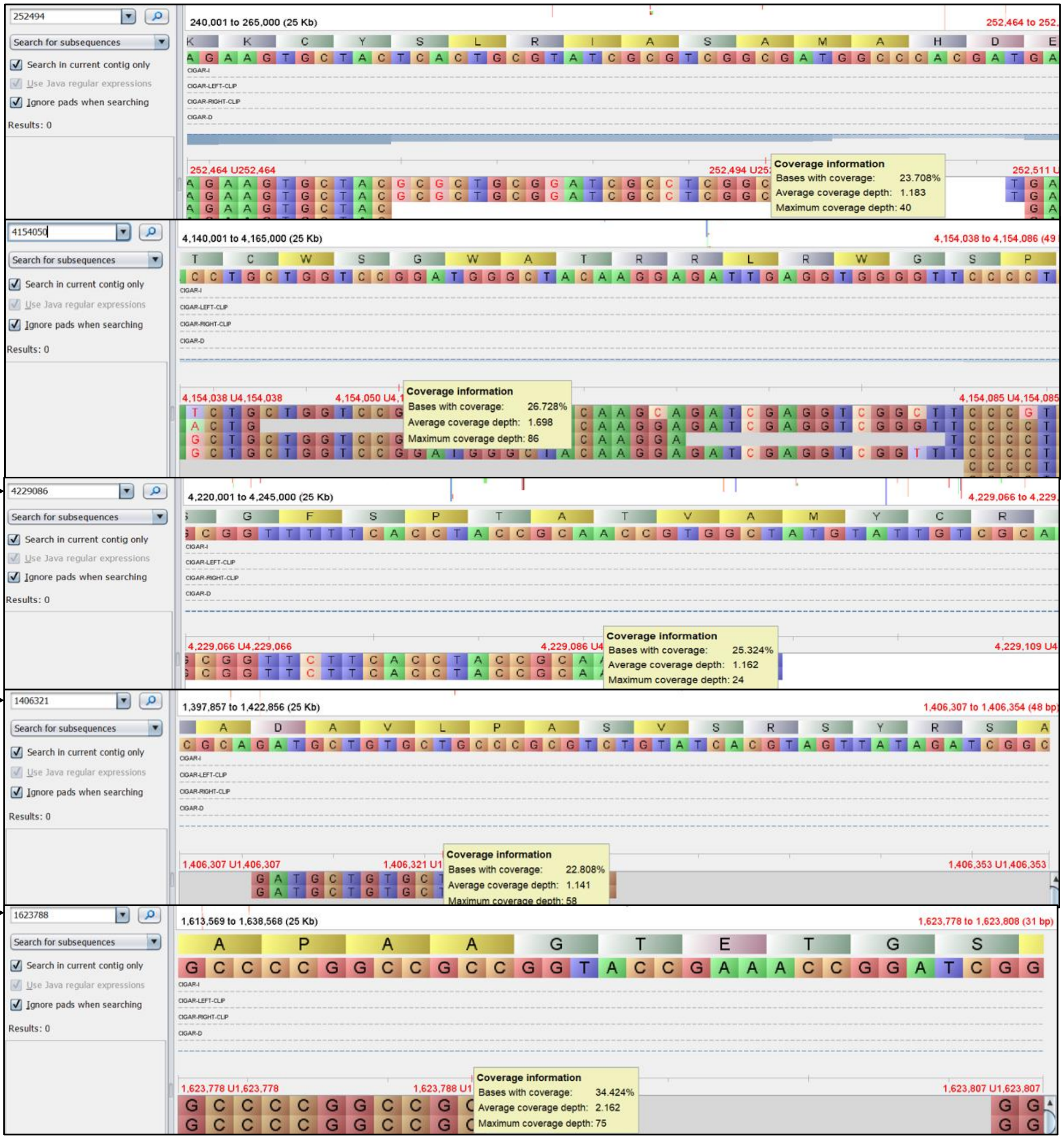

C) Sample ID: KHBAL8- lineage 4.6.2.1 and lineage 1.2.1.2

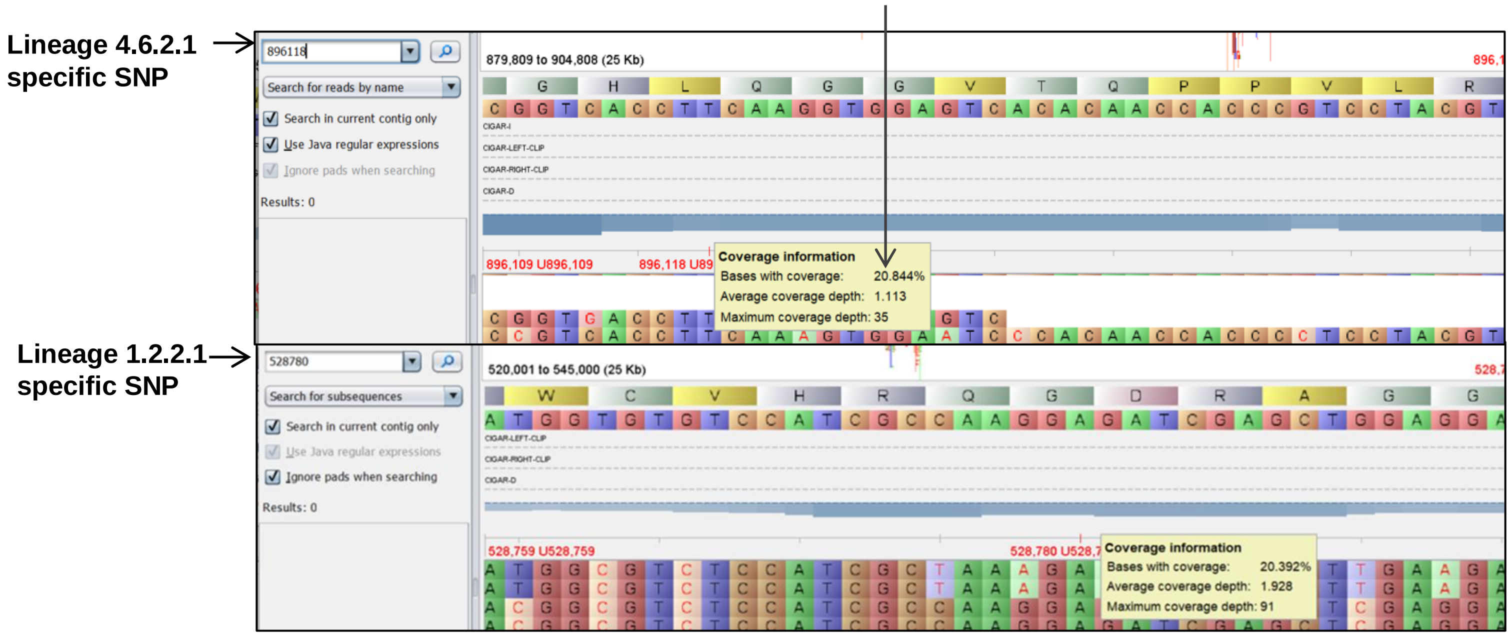

D) Sample ID: KHPus2- lineage 4.6.2.1 and lineage 1.2.1.2

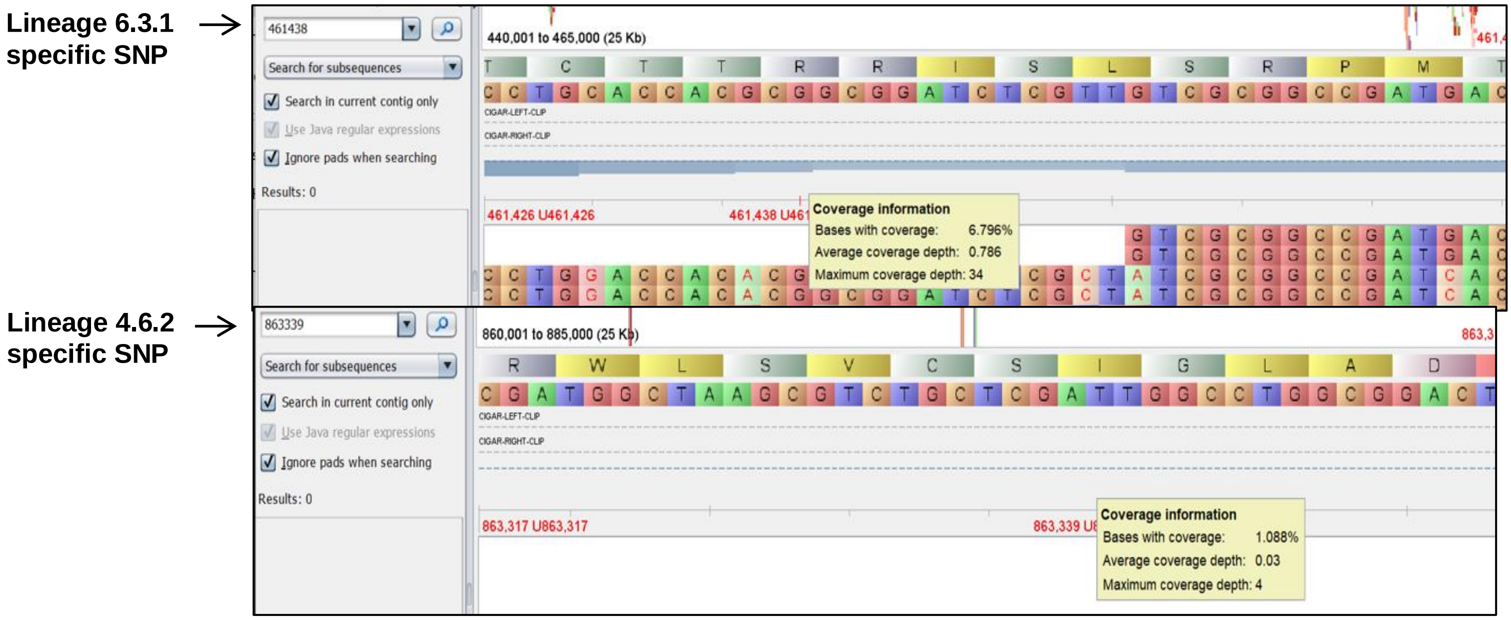

Supplement: ofae320_Supplementary_Data [file ofae320_supplementary_data.zip › supplementary figure 2.pdf]
